# FRAP-CROSS technique: Fracking and Rendezvous-PIERCE for intracalcium crossing in femoropopliteal diffuse calcified occlusions

**DOI:** 10.1186/s42155-025-00626-y

**Published:** 2025-11-27

**Authors:** Takuya Haraguchi, Masanaga Tsujimoto, Ricky Wang-Hei Leung, Yaowen Chang, Yuhei Kasai, Daisuke Hachinohe, Yoshifumi Kashima

**Affiliations:** 1 Department of Cardiology, Sapporo Cardiovascular Clinic, Sapporo Heart Center, Asia Medical Group, Sapporo, Hokkaido Japan; 2 Cardiology Division, Department of Medicine, Li Ka Shing Faculty of Medicine, The University of Hong Kong, Hong Kong, China; 3 Department of Interventional Medicine, The First Hospital of Lanzhou University, Lanzhou, China; 4 Department of Interventional Cardiology, Oita Cardiovascular Hospital, Oita, Japan

**Keywords:** Chronic total occlusion, Calcification, Recanalization, Fracking, Atherectomy, Needle, Peripheral artery disease, Endovascular treatment

## Abstract

**Background:**

Femoropopliteal diffuse calcified occlusions (FPDCOs) are challenging, especially in high-bleeding-risk patients for whom a stentless strategy is preferred. We introduce FRAP-CROSS, combining Fracking and Rendezvous-PIERCE, to achieve intracalcium guidewire crossing and facilitate stentless revascularization.

**Materials and methods:**

When bidirectional intracalcium wiring fails across dense calcification in FPDCO, FRAP-CROSS is applied. Fracking is initially performed by inserting a 20-gauge metal needle into a guidewire-uncrossable plaque and applying hydraulic pressure to create microfractures, facilitating subsequent guidewire crossing. If device tracking remains unsuccessful after guidewire passage, Rendezvous-PIERCE is employed. An 18-gauge needle is advanced toward the intralesional guidewire tip, and the guidewire is externalized through the needle (Needle Rendezvous). A 20-gauge needle is then advanced over the externalized guidewire to create a lumen within the calcification (inner PIERCE). After successful all-intracalcium crossing, balloon angioplasty is performed. Inadequate expansion prompts additional Fracking alone or with Jetstream atherectomy (JET-Frack). Drug-coated balloon (DCB) angioplasty completes the stentless strategy.

**Results:**

A 90-year-old man at high bleeding risk with bilateral FPDCOs underwent FRAP-CROSS. The right limb required three Fracking and two Rendezvous-PIERCE; the left required four Fracking and two Rendezvous-PIERCE, respectively, with adjunctive JET-Frack. Following DCB-based stentless treatment, final angiography and intravascular ultrasound confirmed adequate luminal expansion and blood flow in both limbs, without major complications.

**Conclusion:**

FRAP-CROSS provides a practical approach to achieve all-intracalcium guidewire crossing and stentless revascularization in complex FPDCOs. Further studies should assess its safety and long-term outcomes.

**Graphical Abstract:**

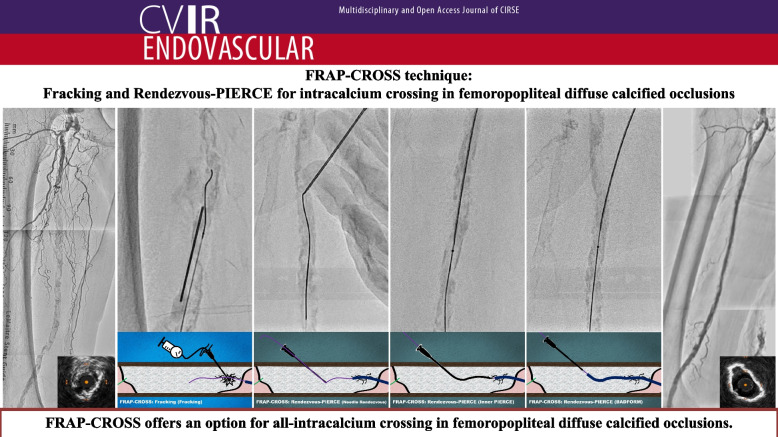

**Supplementary Information:**

The online version contains supplementary material available at 10.1186/s42155-025-00626-y.

## Introduction

Femoropopliteal diffuse calcified occlusions (FPDCOs) remain among the most challenging lesions in endovascular therapy. Conventional high tip-load guidewires and re-entry devices often fail to achieve recanalization; in such cases, subintimal strategies using scaffolds may be necessary. To overcome these limitations, needle-based calcium-modification techniques have evolved. The Fracking technique, which creates hydraulic microfractures within deep calcification, has been reported to facilitate luminal expansion during balloon angioplasty [1, 2]. For lesions in which device passage is not feasible, the needle rendezvous technique, which externalizes a guidewire via a percutaneously introduced needle, can be effective [3]. Moreover, Rendezvous-PIERCE is a plaque-modification technique in which a needle is advanced along the externalized guidewire to directly disrupt calcified plaque [4].

The FRAP-CROSS technique combines Fracking and Rendezvous-PIERCE to create intracalcium microchannels within the calcified occlusion, thereby facilitating guidewire passage and subsequent device delivery while enabling a stentless treatment strategy. We present FRAP-CROSS as a novel intracalcium wiring strategy for FPDCOs.

## Methods

For intracalcium recanalization in FPDCOs, a bidirectional approach is initially attempted using the highest tip-loaded guidewire (CROSSLEAD Penetration 0.018, 60 g; Asahi Intecc, Japan) to perform standard intracalcium wiring. In cases where the guidewire fails to cross the densely calcified segment, the FRAP-CROSS technique is employed. This method employs Fracking to create microchannels in calcified lesions, enabling guidewire passage, followed by Rendezvous-PIERCE to facilitate device tracking and achieve complete intracalcium crossing. Ideal candidates include the following: (1) Diffuse, high-density calcified occlusions of the femoropopliteal segment in which conventional high tip-load guidewires and standard re-entry techniques fail or are predicted to fail, (2) stent-intolerant or high-bleeding-risk patients unsuitable for long-term antiplatelet or anticoagulant therapy, candidates for a stentless strategy; and (3) cases in which feasible access allows precise needle control (antegrade and/or retrograde). Cases with limited hemostatic reserve in whom a high procedural bleeding risk is anticipated, such as those with severe thrombocytopenia or a bleeding diathesis, should be avoided.

### Fracking

**Fig. 1:**
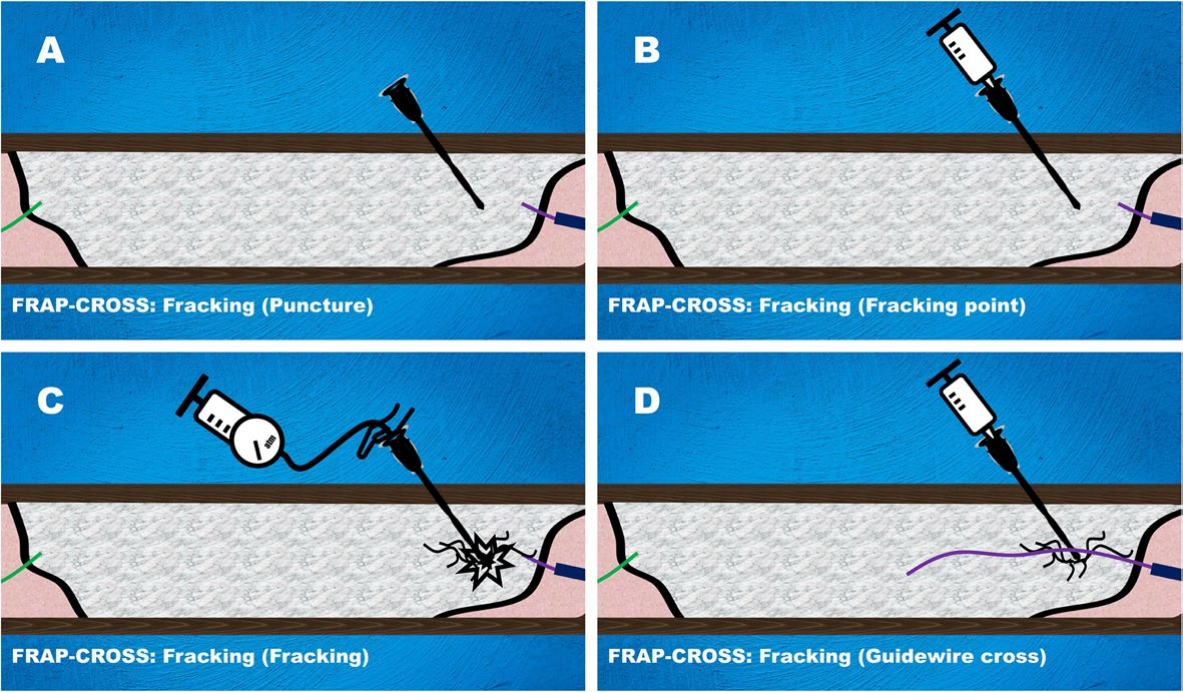
Fracking in FRAP-CROSS. **A** Controlled drilling of a 20-gauge metal needle into the guidewire-uncrossable calcified segment. **B** Fracking-point identification via plunger resistance after attaching a partially saline-filled 3-mL syringe to the needle. **C** Fracking via indeflator-mediated hydraulic pressurization to an abrupt pressure-drop endpoint, creating intracalcium microfracture. **D** Reattempted guidewire passage after syringe reattachment with confirmed Fracking success

Fracking is performed to create microfractures (functionally equivalent to microchannels) within the native high-density calcification, which cannot be penetrated with the highest tip-loaded guidewire (Fig. 1).

1. A 20-gauge metal needle prepared by removing the plastic outer sheath from an 18-gauge needle (Terumo, Japan) or a 21-gauge metal needle (micro-puncture introducer set, Cook Medical, USA) is advanced in a controlled drilling manner into the guidewire-uncrossable calcified segment (Fig. 1A).
2. A 3-mL syringe partially filled with saline is attached to the needle, and the plunger is depressed. If resistance prevents plunger advancement, the needle tip is presumed to be embedded in high-density calcium, designated as the Fracking point (Fig. 1B).
3. The syringe is then replaced with an indeflator containing either saline or a 1:1 mixture of contrast and saline. The hydraulic pressure is gradually applied until a sudden pressure drop indicates microfracture within deep calcium (Fracking) (Fig. 1C).
4. The syringe is reattached to confirm plunger advancement. If the plunger can be pushed, Fracking has succeeded. If resistance persists, indicating an unsuccessful calcium fracture, Fracking is repeated.
5. After successful Fracking, guidewire passage is reattempted (Fig. 1D). If guidewire crossing remains unsuccessful, additional Fracking is sequentially performed at other resistant segments until successful guidewire crossing is achieved.

### Rendezvous-PIERCE

**Fig. 2:**
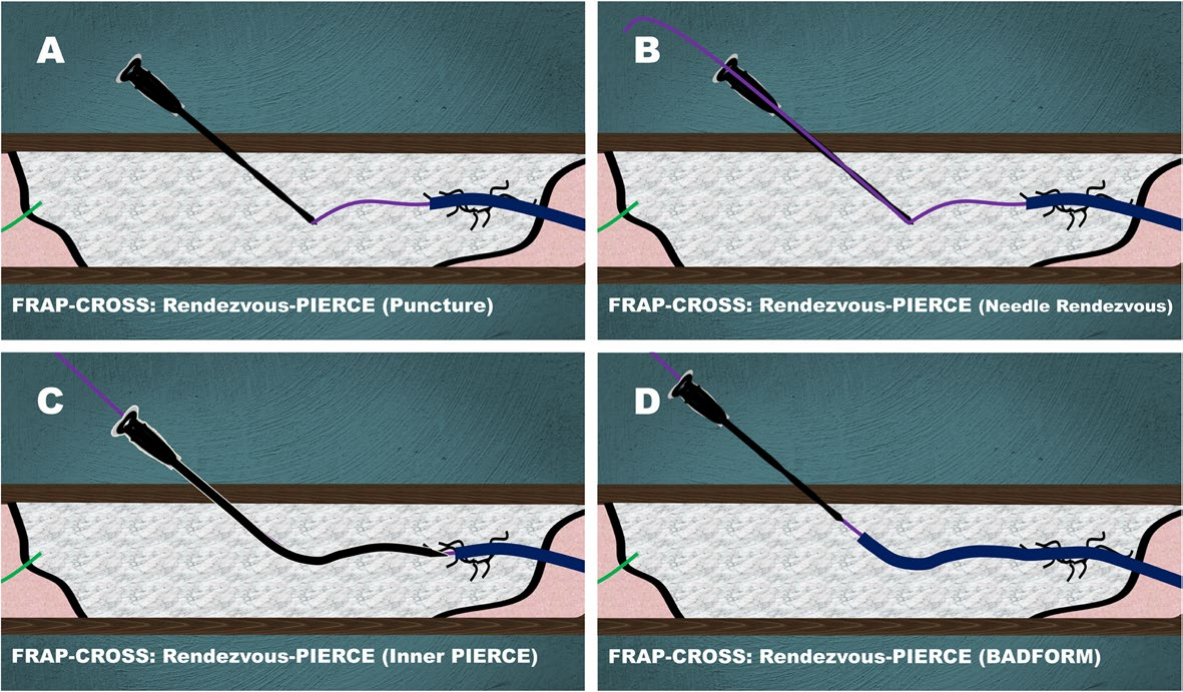
Rendezvous-PIERCE in FRAP-CROSS. **A** Advancement of an 18-gauge needle to the guidewire tip within the calcified occlusion for in-plaque needle rendezvous under multi-angle fluoroscopy. **B** Needle rendezvous for guidewire externalization within the occlusive lesion. **C** Inner PIERCE for needle insertion along the externalized guidewire, creating a lumen within the calcification. **D** BADFORM for microcatheter advancement through the needle-created lumen

If the guidewire successfully crosses but the calcified segment impedes microcatheter or small balloon tracking, Rendezvous-PIERCE is performed (Fig. 2).

1. An 18-gauge needle (Terumo, Japan) is advanced to the guidewire tip within the calcified occlusion to enable in-plaque needle rendezvous under multi-angle fluoroscopy to align both direction and depth (Fig. 2A).
2. After the needle reaches the guidewire, the guidewire is advanced through the needle lumen and externalized (needle rendezvous) (Fig. 2B).
3. A 20-gauge 10-cm or 20-cm needle (Medikit Corp., Tokyo, Japan), selected according to target length, is then advanced along the externalized guidewire into the calcified segment to create a lumen (Inner PIERCE) (Fig. 2C).
4. The microcatheter is forcefully pulled to cross the lesion over the externalized guidewire using the Balloon deployment using FORcible Manner (BADFORM) technique [5], and wiring is continued after successful microcatheter crossing (Fig. 2D).

Following successful FRAP-CROSS, balloon angioplasty is performed. In cases of inadequate vessel expansion, additional plaque modification is conducted using Fracking alone or in combination with Jetstream atherectomy (JET-Frack) to address both superficial and deep calcification. Once sufficient luminal gain is confirmed by angiography and intravascular ultrasound (IVUS), the procedure is completed with a stentless strategy using a drug-coated balloon (DCB).

## Results

A 90-year-old male with bilateral FPDCOs presented with severe intermittent claudication. Due to his high bleeding risk, revascularization was performed using FRAP-CROSS for stentless treatment.

On the right side, bidirectional wiring failed to cross the right FPDCO with a calcified lesion length of 270 mm (occlusion length 220 mm). For intracalcium crossing, Fracking was performed three times and Rendezvous-PIERCE twice to complete the intracalcium crossing. Lesion dilation was performed using 4.0 and 6.0 × 300-mm noncompliant balloons (SHIDEN HP, Kaneka, Japan). Additional Fracking was conducted at two underexpanded segments to achieve complete balloon expansion. Finally, two 6.0-mm DCBs were used. The final angiogram and IVUS confirmed sufficient flow and luminal area.

On the left side, the FPDCO with a calcified lesion length of 380 mm (occlusion length 310 mm) was successfully crossed with FRAP-CROSS, involving four Fracking and two Rendezvous-PIERCE attempts. Unexpanded calcifications were modified with Jetstream atherectomy (2.1/3.0 mm; Boston Scientific, Marlborough, USA) to modify superficial calcification, followed by pre-dilation with a 6.0 × 300 mm noncompliant balloon. IVUS demonstrated persistent luminal narrowing due to deep calcification; therefore, three additional Fracking procedures were performed, resulting in sufficient luminal expansion. The entire lesion was then dilated with a 6.0-mm DCB. Final angiography and IVUS demonstrated the complete success of stentless revascularization with JET-Frack.

**Fig. 3:**
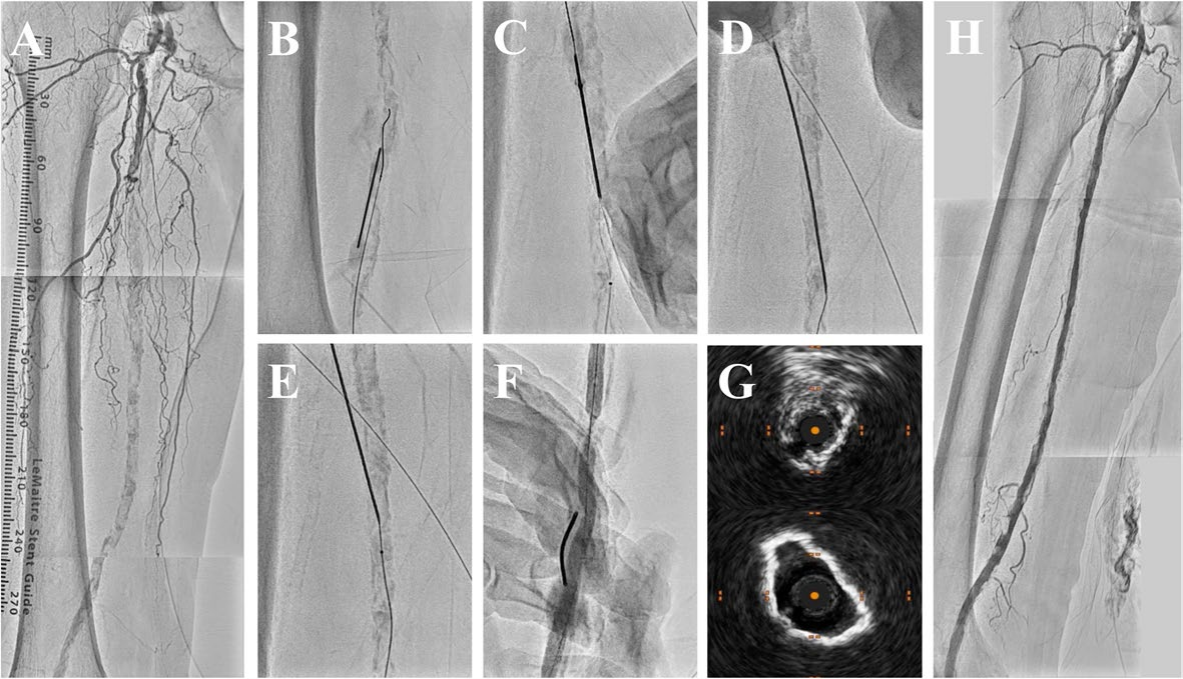
FRAP-CROSS for right femoropopliteal diffuse calcified occlusion. **A** Baseline angiogram. **B** Successful guidewire crossing after Fracking. **C** Needle rendezvous. **D** Rendezvous-PIERCE. **E** BADFORM. **F** Additional Fracking for modification of underexpanded calcified segments. **G** IVUS showing the change in minimum lumen area before (upper panel) and after (lower panel) balloon angioplasty. **H** Final angiogram

**Fig. 4:**
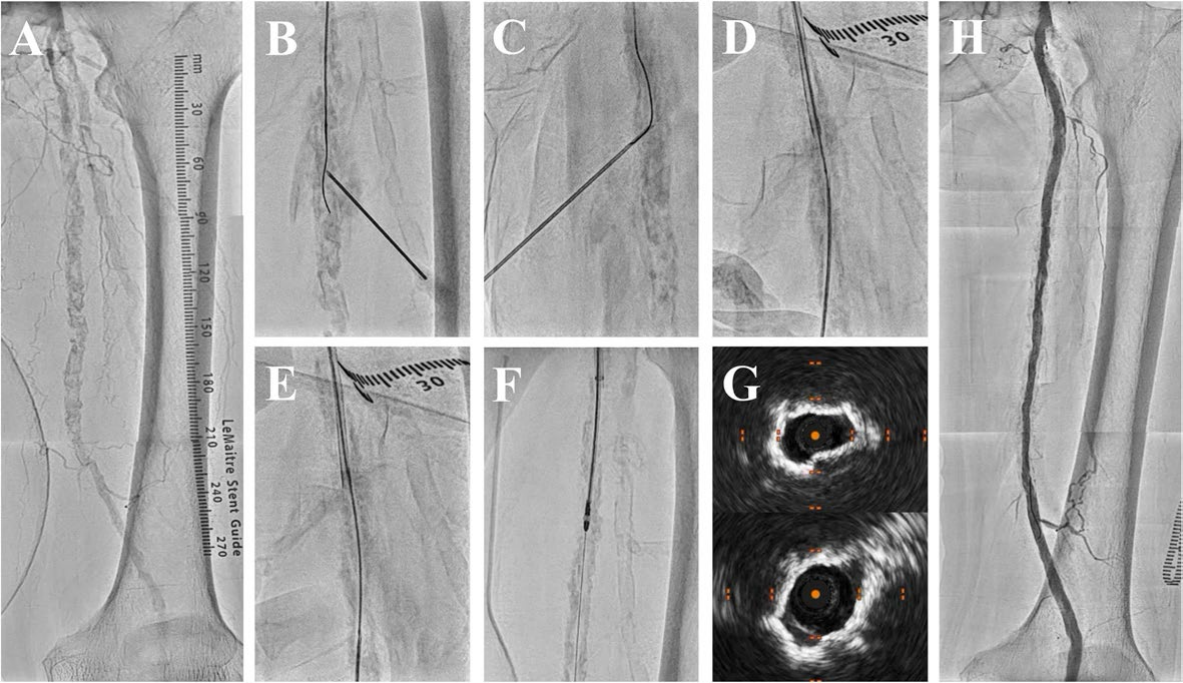
FRAP-CROSS with JET-Frack for left femoropopliteal diffuse calcified occlusion. **A** Baseline angiogram. **B** Successful guidewire crossing after Fracking. **C** Needle rendezvous. **D** Rendezvous-PIERCE. **E** BADFORM. **F** Jetstream atherectomy modifying diffuse superficial calcifications. **G** IVUS showing the change in minimum lumen area before (upper panel) and after (lower panel) additional Fracking following Jetstream. **H** Final angiogram

Intra-procedurally, an initial bolus of unfractionated heparin (5000 IU) was administered, with additional doses titrated to maintain an activated clotting time ≥ 250 s. Only minor hematomas at FRAP-CROSS puncture sites were observed, and no clinically significant bleeding occurred during the periprocedural period. No flow-limiting dissection or distal embolization was observed on angiography, and IVUS showed no significant flap formation. Procedural time, fluoroscopy time, and contrast volume were 176 min, 97 min, and 120 mL for the right limb and 140 min, 70 min, and 161 mL for the left limb, respectively. Figures 3 and 4 and Supplementary Videos 1 and 2 illustrate these techniques in the present procedures.

## Discussion

Fracking has been described as a technique in which balloon inflation densifies deep-seated calcification, followed by hydraulic cracking and subsequent balloon angioplasty to compress the fractured calcium, thereby achieving larger luminal gain [1]. In native high-density calcified lesions, where even a high tip-load guidewire cannot cross, as in the present case, Fracking can create microfractures within the calcified occlusion without balloon support, thereby facilitating guidewire passage through previously uncrossable segments. Consistent with this concept, an ex vivo micro-computed tomography analysis has demonstrated microfracture formation within dense calcium produced by Fracking, without arterial injury or dissection, in the absence of balloon support [6]. Rendezvous-PIERCE has also been reported to reduce procedural time by intravascular modification of calcified plaque using a needle, compared to needle rendezvous for guidewire externalization and subsequent device passage [3]. As FRAP-CROSS may require multiple percutaneous punctures, careful attention must be paid to bleeding complications. In our experience, a combination of intravascular balloon tamponade and external compression using gauze and elastic bandages may prevent hemorrhage. As intravascular pressure is lower in the distal than the proximal segments of the occlusions, applying this technique distally or when targeting the retrograde guidewire may further reduce the risk of significant bleeding complications. These observations position FRAP-CROSS as a practical intracalcium wiring adjunct when subintimal scaffolding would otherwise be required.

### Limitations

This technique requires advanced needle manipulation skills and expertise in both Fracking and Rendezvous-PIERCE. Although stenting was avoided in the present case, scaffold implantation may be necessary when luminal gain is insufficient. Given the need for multiple needle punctures under systemic heparinization, careful patient selection is essential, particularly in those with limited hemostatic reserve. This report is based on a single case, and verification of long-term patency and safety remains a future challenge.

## Conclusions

The FRAP-CROSS technique offers a promising option for achieving all-intracalcium crossing in FPDCOs. Further investigation is needed to determine its applicability across a broader range of lesion morphologies and clinical settings.

## Supplementary Information

Additional file 1: Supplementary Video 1. FRAP-CROSS for right FPDCO.

Additional file 2: Supplementary Video 2. FRAP-CROSS with JET-Frack for left FPDCO.

## Data Availability

The data are available from the corresponding author upon reasonable request.

## Abbreviations

DCB: Drug-coated balloon
FPDCO: Femoropopliteal diffuse calcified occlusion
IVUS: Intravascular ultrasound
